# Interactions between malaria and HIV infections in pregnant women: a first report of the magnitude, clinical and laboratory features, and predictive factors in Kinshasa, the Democratic Republic of Congo

**DOI:** 10.1186/s12936-015-0598-2

**Published:** 2015-02-18

**Authors:** Roger D Wumba, Josué Zanga, Michel N Aloni, Kennedy Mbanzulu, Aimé Kahindo, Madone N Mandina, Mathilde B Ekila, Oussama Mouri, Eric Kendjo

**Affiliations:** Department of Tropical Medicine, Infectious and Parasitic Diseases, Department of Parasitology, University Clinic of Kinshasa, Faculty of Medicine, University of Kinshasa, Kinshasa, Congo; Department of Pediatrics, University Hospital of Kinshasa, Faculty of Medicine, University of Kinshas, Kinshasa, the Democratic Republic of Congo; Department of Internal Medicine, University Hospital of Kinshasa, Faculty of Medicine, University of Kinshasa, Kinshasa, the Democratic Republic of Congo; Centre Nationale de Référence du Paludisme, AP-HP, CHU Pitie Salpêtrière-Charles Foix, 47, boulevard de l’Hôpital, 75651 Paris, Cedex 13 France; Laboratory of Parasitology and Mycology, Pitié Salpêtrière Hospital, Public Assistance-Hospitals of Paris, Pierre and Marie Curie University, Paris, France; Department of Parasitology, Mycology and Tropical Medicine, Faculty of Medicine, University of Health Sciences, Libreville, Gabon; Institute of Tropical Medicine, University of Tubingen, Tubingen, Germany

**Keywords:** Malaria, HIV, Low birth weight, Pregnant women, Kinshasa, the Democratic Republic of Congo, Africa

## Abstract

**Background:**

HIV and malaria are among the leading causes of morbidity and mortality during pregnancy in Africa. However, data from Congolese pregnant women are lacking. The aim of the study was to determine the magnitude, predictive factors, clinical, biologic and anthropometric consequences of malaria infection, HIV infection, and interactions between malaria and HIV infections in pregnant women.

**Methods:**

A cross-sectional study was conducted among pregnant women admitted and followed up at Camp Kokolo Military Hospital from 2009 to 2012 in Kinshasa, the Democratic Republic of Congo. Differences in means between malaria-positive and malaria-negative cases or between HIV-positive and HIV-negative cases were compared using the Student’s *t*-test or a non-parametric test, if appropriate. Categorical variables were compared using the Chi-square or Fisher’s exact test, if appropriate. Backward multivariable analysis was used to evaluate the potential risk factors of malaria and HIV infections. The odds ratios with their 95% confidence interval (95% CI) were estimated to measure the strengths of the associations. Analyses resulting in values of P < 0.05 were considered significant.

**Results:**

A malaria infection was detected in 246/332 (74.1%) pregnant women, and 31.9% were anaemic. Overall, 7.5% (25/332) of mothers were infected by HIV, with a median CD4 count of 375 (191; 669) cells/μL. The mean (±SD) birth weight was 2,613 ± 227 g, with 35.7% of newborns weighing less than 2,500 g (low birth weight). Low birth weight, parity and occupation were significantly different between malaria-infected and uninfected women in adjusted models. However, fever, anemia, placenta previa, marital status and district of residence were significantly associated to HIV infection.

**Conclusion:**

The prevalence of malaria infection was high in pregnant women attending the antenatal facilities or hospitalized and increased when associated with HIV infection.

## Background

Malaria is well established as a major global health problem worldwide [1,2]. Malaria, Human Immunodeficiency Virus (HIV) and tuberculosis are among the three most important global health problems of developing countries. Both of them together, cause more than four million deaths per year [2]. The co-infection exists in many parts of the world. This is particularly true in sub-Saharan Africa, where an estimated 40 million people are living with HIV and more than 350 million episodes of malaria occur yearly [1]. Malaria and HIV infections are also the most deleterious conditions in sub-Saharan African pregnant women, in terms of the morbidity and mortality they cause in mothers and their newborns [3-9].

In the DRC, malaria is reported to be the principal cause of morbidity and mortality, especially among vulnerable population like pregnant women and children less than five years of age. Close to 70% of all outpatient health care visits and an average of 30% of hospital admissions are malaria-related [10]. A recent study in the DRC based on Demographic and Health Survey (DHS) in 2007 showed an increase risk for malaria in certain populations and regions of the DRC with higher prevalence in rural area than urban [11]. In 2003, UNAIDS estimated that the prevalence of HIV in adult in DRC was 4.2% and 1.1 million people were living with HIV/AIDS [12]. However, this prevalence among pregnant women was ranged from 2.7 to 5.4% in 1999 in urban area and 8.5% in rural area [12].

Understanding the epidemiology of malaria and HIV during pregnancy is important for deciding the best control strategies. Unfortunately, vital registration systems in sub-Saharan African countries have low coverage and do not produce accurate estimation of this co-infection. Health workers in the Congolese Army are unable to identify high or risk factors for both malaria and HIV infections so as to tailor interventions and do effective health monitoring. Moreover, pregnant women married to soldiers and single soldiers are habitually facing war-related poverty and human movements. Therefore, the study aims were to determine the magnitude, predictive factors, clinical, biologic and anthropometric consequences of malaria infection, HIV infection, and interactions between malaria and HIV infections in pregnant women.

## Methods

### Study design

A prospective study for the prevalence of malaria, HIV infection, HIV co-infection, and low birth weight from pregnancy to delivery as well as a comparative study of health consequences at the delivery was conducted from 2009 to 2012.

### Study site

The Camp Kokolo Military Hospital (HMRK) was the study setting and is the largest public hospital in the country, situated at the western part of Kinshasa, the capital city of DRC. The town covers an area of 9,965.21 km^2^ with an estimated total population of 7,411,989 of whom more than 50% are female [13]. The HMRK provides antenatal services and has a well-established prevention of mother-to-child transmission of HIV (PMTCT) programme.

### Study population

**Figure 1:**
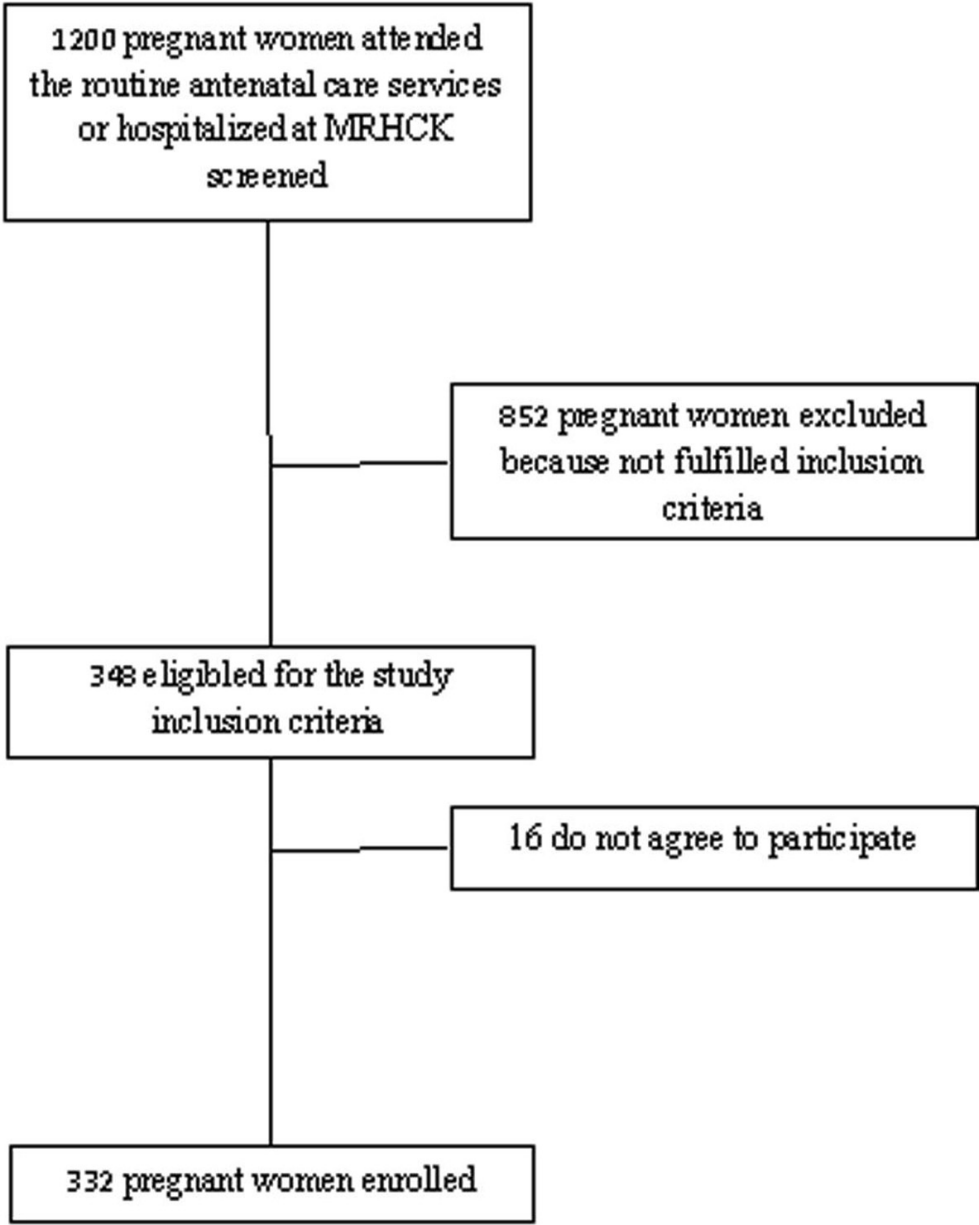
**Study profile.**

All pregnant women who were suspected of malaria and followed up in routine antenatal care service at HMRK or hospitalized in the same hospital with at least one antenatal visit between 2009 and 2012 were included to this study (Figure 1).

### Ethics statement

The institutional review boards and the Ethics Committee of the University Protestant of Kinshasa approved the protocol of the study (CEUPC 0018), which was conducted in compliance with the principles of the Helsinki Declaration II. Patients were informed about the research purposes and gave written informed consent. The Ethics Committee of the Université Protestante du Congo (UPC) approved the consent procedure. The guidance of this study was in the context of the routine diagnosis and of normal medical care. Limited epidemiological, clinical and biological information were collected anonymized. Blood samples collection were also performed in the context of the routine diagnosis and of normal medical care for which the department of Tropical Medicine was granted the agreement N/Réf: 007/UPC/SGAC/MM/AB/2009 from the Medical faculty of UPC and the Faculty of Medicine of the University of Kinshasa.

### Data collection

Mothers were interviewed to obtain socio-demographic, biographic, and clinical variables. These pregnant women were tested using both blood films (thin and thick blood smear) and polymerase chain reaction (PCR) for the presence of *Plasmodium*, responsible for malaria cases. Study participants who were positive for malaria were treated. All mothers were provided a standard counseling and were systematically screened for the presence of HIV antibody by using Determine HIV 1/2 rapid test kits (Abbott Diagnostic Division, Hoofddorp, The Netherlands). At the delivery, birth weights of newborns were recorded using standard methods.

### Definitions

The demographic details recorded were: age on admission classified as follows: < 20 years, 20–24 and ≥ 25 years, marital status (married spouses of soldiers *vs.* single women as parents of soldiers), occupation (housewives without income *vs.* others with income), and the district of residence (urban for Funa, semi-urban for Amba, and Rural for Lukunga). The laboratory variables were: haemoglobin concentration (g/dL) and parasitaemia (parasites/μL). Clinical variables were: history of fever, body aches defines as the combination of asthenia and muscle pain, headaches, nausea and vomiting, bleeding, respiratory tract infection (rhinorrhoea and cough), placenta previa, digestive disorders with gastritis, constipation, diarrhoea and Salmonella infection.

### Statistical analysis

The variables were summarized as frequencies, means (standard deviation = SD), medians (interquartile range = IQR). Differences in means between malaria-positive and malaria-negative cases or between HIV-positive and HIV-negative cases were compared using the Student’s *t*-test or a non-parametric test, if appropriate. Categorical variables were compared using the Chi-square or Fisher’s exact test, if appropriate. Backward multivariable analysis was used to evaluate the potential risk factors of malaria and HIV infections. The odds ratios with their 95% confidence interval (95% CI) were estimated to measure the strengths of the associations. Analyses resulting in values of P < 0.05 were considered significant. All reported p-values were two-tailed using Stata, version 12 *(Stata Corp, College station, Texas)*.

## Results

### Characteristics of the study population

Over the study period, a total of 1,200 pregnant women attended the routine antenatal care services or hospitalized at MRHCK were screened, out of whom 348 were eligible for the study. Out of these, 332 agreed to participate (95.4%) and were tested for malaria and HIV infections (Figure 1). Most of the women that participated in the study were housewives (88.8%) and were married (84.6%). The mean (±SD) age of participants was 28.6 ± 6.1 years and the median number of previous pregnancies, number of children and abortion was three (range 2–5), two (range 2–4) and two (range 1–3), respectively. Additionally, around 40% were in the first trimester of pregnancy. Overall, the mean (±SD) birth weight was 2613 ± 227 g, with 35.7% of newborns weighed less than 2,500 g.

### Clinical examination and laboratory assessments of malaria in pregnant women admitted to the study

The overall malaria parasite proportion was 74.1% (246/332) and *Plasmodium falciparum* was the only species detected in the positive malaria slide of pregnant women whose peripheral blood smears were tested. Of the 332 pregnant women whose hemoglobin levels were analyzed at enrolment, 31.9% (106/332) were anaemic (Hb  <  11.0 g/dL). Out of the 332 pregnant women who were interviewed, 37.9% (126/332) had fever, 14.9% (49/332) had headache and 3.5% (38/332) complained of general body weakness whereas 39.8% (132/332) had respiratory tract infection. Other symptoms included nausea 7.5% (25/332), digestive disorder 10.5 (35/332) and bleeding 6.9% (23/332). The geometric parasite density and the 95% confidence interval (CI) in patients with positive slide were 3383 (IQR = 2038-5618) parasites/μL.

### Predictive factors associated with incident malaria infection

Table 1 summarizes the risk factors associated with malaria infection in pregnant women using univariate analysis. There was a significant association between urban/Funa district, lack of income in housewives, higher parity with ≥3 children, and incident malaria infection. Low birth weight was more prevalent in presence of malaria than in absence of malaria, the difference being statistically significant. In contrast, the rest of variables of interest were not associated (p > 0.05) with malaria presence. In multivariate analysis, after adjusting for confounder residence, lack of income in housewives (adjusted odds ratio = 3.3 95%CI 1.6 to 6.6; p < 0.001) and higher parity (adjusted odds ratio = 1.9 95%CI 1.1 to 3.3; p = 0.04) remained significantly associated with incident malaria infection.

**Table 1:** **Demographic, clinical and laboratory test characteristics and adjusted odds ratios measuring the associations between malaria infection and risk factors in pregnant women** ^**1, 2, 3**^

**Characteristics**	**Malaria infected pregnant women**				
	**Overall n (%) (N = 332)**	**Malaria-positive (%) (N = 246)**	**COR (95% CI)**	**AOR (95% CI)**	**P**
**Maternal age (years)**					
<20	21 (6.4%)	14 (66.7%)	1		0.6
20–24	75 (22.7%)	58 (77.3%)	1.7 (0.6-4.9)		
> = 25	234 (70.9%)	173 (73.9%)	1.4 (0.5-3.7)		
**Number of previous pregnancies**					
Primigravidae	77 (23.3%)	56 (72.7%)	1		
Multigravidae	253 (76.7%)	189 (74.7%)	0.8 (0.4-1.4)		0.3
**Number of children**					
<3	182 (56.2%)	126 (69.2%)	1	1	
≥3	142 (43.8%)	113 (79.6%)	1.7 (1.1-2.9)	1.9 (1.1-3.3)	0.04
**Abortion**					
No abortion	97 (29.2%)	70 (72.2%)	1		
Abortion	235 (70.8%)	176 (74.9%)	1.2 (0.7-2.0)		0.6
**Trimester of visit**					
First-trimester	163 (49.1%)	122 (74.8%)	1		0.7
Second-trimester	88 (26.5%)	65 (73.9%)	1.2 (0.7-2.4)		
Third-trimester	81 (24.4%)	59 (72.8%)	1.1 (0.6-2.3)		
**District of Kinshasa**					
Semi-urban for Amba	21 (6.3%)	13 (61.9%)	1		0.01
Urban for Funa	219 (66.0%)	173 (79.0%)	2.3 (1.2-5.9)		
Rural for Lukunga	92 (27.7%)	60 (65.2%)	1.2 (0.4-3.1)		
**Occupation**					
Others with income	37 (11.2%)	19 (51.4%)	1	1	
Housewives without income	294 (88.8%)	226 (76.9%)	3.1 (1.6-6.3)	3.3 (1.5-6.5)	0.005
**Marital status**					
Single women as parents of soldiers	50 (15.4%)		1		
Married spouses of soldiers	275 (84.6%)	209 (76.0%)	1.8 (0.9-3.4)		0.07
**Low Birth Weight (LBW)**					
≥2500 g	213 (64.3%)				
<2500 g	118 (35.7%)	113 (44.9%)			<0.001
***Examination findings***					
Anaemia (haemoglobin < 11 g/dL), %	106 (31.9%)	76 (71.7%)	0.5 (0.4-1.4)		
Fever	123 (38.0%)	92 (73.0%)	0.9 (0.6-1.5)		0.7
Body aches	38 (11.5%)	25 (65.8%)	0.6 (0.3-1.3)		0.2
Headaches	49 (18.8%)	37 (75.5%)	1.1 (0.5-2.2)		0.8
Digestive disorders	8 (2.4%)	6 (75.0%)	1.1 (0.2-5.3)		0.9
Nausea and vomiting	25 (7.5%)	18 (72.0%)	0.9 (0.3-2.2)		0.8
Respiratory infection	132 (39.8%)	9 (75.0%)	0.8 (0.5-1.4)		0.6
Bleeding	23 (6.9%)	18 (78.3%)	1.3 (0.4-3.6)		0.6
Urinary tract infection	223 (67.2%)	156 (70.0%)	0.5 (0.3-0.8)	0.5 (0.3-0.9)	0.04
Gastritis	24 (7.2%)	18 (75.0%)	1.1 (0.4-2.7)		0.9

^1^CI = confidence interval; COR = Crude odds ratios; AOR = Adjusted odds ratios. ^2^Factors included in the logistic regression model were: age, marital status, occupation, urinary tract infection and HIV status. ^3^All P-values were estimated through Wald tests in adjusted logistic regression models. The model included both Malaria-infected and uninfected pregnant women, adjusting for other predictors.

### Prevalence of HIV-infection

**Figure 2:**
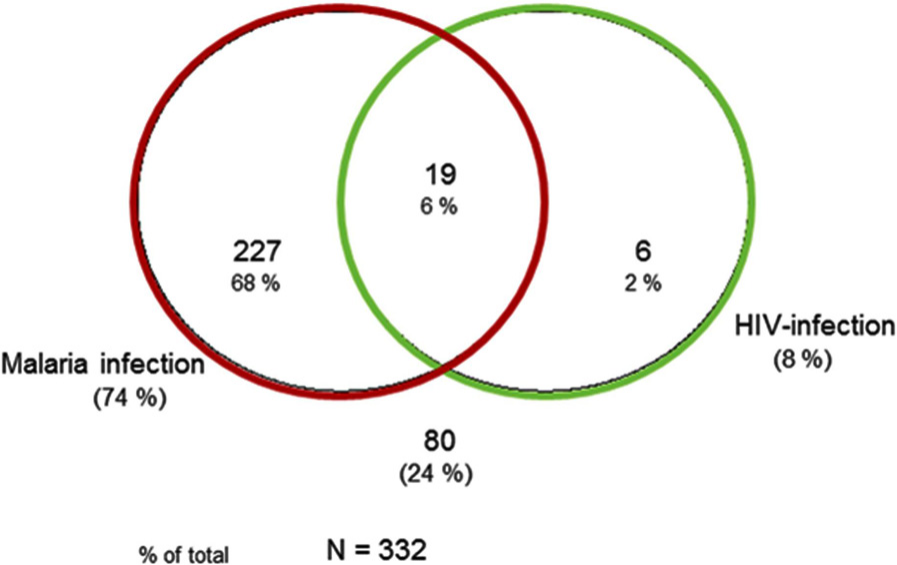
**Venn diagram showing inter-relationship between malaria and HIV infections.**

Figure 2 shows the interrelationship between malaria infection and HIV-infection. Of the 332 patients enrolled, 7.5% (25/332) were HIV-infected, with a median CD4 count of 375 (IQR = 191–669) cells/μL. Nineteen women (6%) were both infected by malaria and HIV and six (2%) were only infected by HIV. Table 2 summarizes the levels of different associations between variables of interest and HIV-infection. Only single marital status, urban residence, fever, placenta previa, and anaemia were significantly associated with HIV-infection. However, there was no significant difference in rates of other variables of interest between HIV-positive and HIV-negative groups. In multivariate analysis and after adjusting for confounders, placenta previa, fever, anaemia, marital status, and urban residence were significantly and independently associated with the presence of HIV-infection (Table 2).

**Table 2:** **Demographic, clinical and laboratory test characteristics of pregnant women infected with HIV** ^**1, 2, 3, 4, 5**^

	**HIV+(N = 25)**	**HIV-(N = 307)**	**AOR (95% CI)**	**P-value**
***Demographic Characteristics***				
Age in years, mean ± SD	29.4 ± 7.2	28.5 ± 6.0		0.6
<20	20 (6.7%)	1 (4.0%)		
20–24	68 (22.3%)	7 (28.0%)		0.6
> = 25	217 (71.0%)	17 (68.0%)		
LBW (<2500gr)	12 (48.0%)	106 (34.6%)		0.2
Single women as parents of soldiers, (%)	4 (16.0%)	273 (10.8%)	3.5 (1.1-11.2)	0.04
**Occupation, (%)**				
Housewives without income	21 (84.0%)	273 (89.2%)		0.4
**Number of previous pregnancies, (%)**				
Primigravidae	6 (24.0%)	71 (23.3%)		
Multigravidae	19 (76.0%)	234 (76.7%)		0.9
**Number of children, (%)**				
<3	16 (64.0%)	166 (55.5%)		0.4
> = 3	9 (36.0%)	133 (44.5%)		
**Number of abortion, (%)**				
No abortion	10 (40.0%)	87 (28.3%)		
Abortion	15 (60.0%)	220 (71.7%)		0.2
**Trimester of visit, (%)**				
First-trimester	12 (48.0%)	151 (49.2%)		
Second-trimester	8 (32.0%)	80 (26.1%)		0.7
Third-trimester	5 (6.2%)	76 (24.8%)		
**District of Kinshasa, (%)**				
Rural for Lukunga	4 (16.0%)	88 (28.7%)	1	
Semi-urban for Amba	4 (16.0%)	17 (5.5%)	10.6 (1.8-62.7)	
Urban for Funa	17 (68.0%)	202 (65.8%)	2.9 (0.8-10.6)	0.03
***Examination findings***				
Fever, (%)	20 (80.0%)	5 (34.0%)	9.4 (2.9-30.0)	<0.001
Body aches, (%)	1 (4.0%)	37 (12.1%)		0.3
Headaches, (%)	5 (10.2%)	20 (7.1%)		0.4
Digestive disorders, (%)	2 (8.0%)	33 (10.8%)		0.7
Nausea and vomiting, (%)	2 (8.0%)	23 (7.5%)		0.9
Respiratory infection, (%)	12 (48.0%)	120 (39.1%)		0.4
Bleeding, (%)	0	23 (7.5%)		0.2
Placenta previa, (%)	3 (12.0%)	27 (8.8%)	11.9 (1.4-104.5)	<0.0001
Urinary tract infection, (%)	14 (56.0%)	208 (67.8%)		0.2
***Laboratory tests***				
Haemoglobin (g/dL), mean ± SD	11.8 ± 4.8	13.8 ± 4.4		0.04
Anaemia (haemoglobin < 11 g/dL), %^4^	16 (64.0%)	92 (30.0%)	4.1 (1.6-10.8)	<0.001

^1^IQR = interquartile range; SD = standard deviation. ^2^ To compare HIV+ and HIV-, Pearson chi-square tests and Fisher’s exact test were used to compare proportions, ttest and Kruskal-Wallis tests were used to compare continuous variables. ^3^ Sample sizes reported in this table and used for estimation of proportions include subjects with outcome missing. ^4^Factors included in the logistic regression model were: district of residence, marital status, occupation, fever, placenta previa and anaemia. ^5^All P-values were estimated through Wald tests in adjusted logistic regression models. The model included both HIV-infected and uninfected pregnant women, adjusting for other predictors.

### Contributing factors of low birth weight

Except of malaria infection, the rest variables were not associated (P > 0.05) with (Table 3).

**Table 3:** **Demographic, clinical and laboratory test characteristics and adjusted odds ratios measuring the associations between LBW and risk factors in pregnant women**

	**LBW (N = 118)**	NBW* (N = 213)	**COR (95% CI)**	**AOR (95% CI)**	**P-value**
***Demographic Characteristics***					
**Maternal age (years)**					
<20	7 (5.9%)	14 (6.6%)	1		
20–24	25 (21.2%)	50 (23.7%)	0.8(0.3-2.3)		
> = 25	86 (72.9%)	147 (69.7%)	1.2(0.5-3.2)		0.9
**Marital status, (%)**					
Married spouses of soldiers	102 (88.7%)	172 (82.3%)	1.0		
Single women as parents of soldiers	13 (11.3%)	37 (17.7%)	0.7(0.3-1.3)		0.1
**Occupation, (%)**					
Others with income	9 (7.7%)	28 (13.2%)	1		
Housewives without income	108 (92.3%)	185 (89.9%)	0,4(0.1-1.7)		0.1
**Number of previous pregnancies, (%)**					
Primigravidae	32 (27.1%)	45 (21.3%)	1.0		
Multigravidae	86 (72.9%)	166 (78.7%)	0.8(0.5-1.3)		0.2
**Number of children, (%)**					
<3	52 (63.9%)	119 (57.2%)	1.0		
> = 3	53 (49.1%)	89 (42.8%)	1.5(0.9-2.4)		0.6
**Number of abortion, (%)**					
No abortion	34 (28.8%)	62 (29.1%)	1.0		
Abortion	84 (71.2%)	151 (70.9%)	1.1(0.6-1.7)		0.9
**Trimester of visit, (%)**					
First-trimester	63 (53.4%)	100 (47.0%)	1.0		
Second-trimester	31 (26.3%)	57 (26.8%)	0.8(0.5-1.4)		0.4
Third-trimester	24 (20.3%)	56 (26.3%)	0.8(0.5-1.4)		
**District of Kinshasa, (%)**					
Rural for Lukunga	30 (25.4%)	62 (29.1%)	1.0		
Semi-urban for Amba	8 (6.8%)	13 (6.1%)	2.1(0.8-5.5)		0.3
Urban for Funa	80 (67.8%)	132 (64.8%)	1.4(0.8-2.3)		
***Examination findings***			1.1(0.8-1.7)		
Fever, (%)	46 (39.0%)	81 (38.0%)	1.1(0.7-1.7)		
Body aches, (%)	8 (6.8%)	30 (14.1%)	1.2(0.6-2.4)		0.04
Headaches, (%)	15 (12.7%)	34 (16.0%)	1.4(0.8-2.6)		0.4
Digestive disorders, (%)	11 (9.3%)	24 (11.3%)	0.6(0.3-1.3)		0.6
Nausea and vomiting, (%)	5 (4.2%)	20 (9.3%)	1.7(0.8-3.9)		0.09
Respiratory infection, (%)	52 (44.1%)	80 (37.6%)	1.4(0.9-2.1)		0.2
Bleeding, (%)	10 (8.5%)	13 (6.1%)	0.9(0.4-2.3)		0.4
Placenta previa, (%)	13 (11.0%)	17 (8.0%)	1.1(0.5-2.3)		0.3
Urinary tract infection, (%)	70 (59.3%)	151 (70.9%)	0.9(0.5-1.4)		0.03
***Laboratory tests***					
Anaemia (haemoglobin < 11 g/dL), (%^4^)	35 (29.7%)	73 (34.3%)	1.1(0.6-1.7)		0.3
**Outcome**					
Malaria infection, (%)	111 (94.1%)	134 (62.9%)	7.0(3.3-14.5)	8.7 (3.8-19.7)	<0.001

*NBW = normal birth weight.

### Roles of interactions between malaria and HIV infections on variables of interest

Table 4 presents the comparisons of rates of variables of interest across malaria alone, HIV-infection alone, malaria + HIV-infections, and non-infected groups. The highest proportions of low birth weight, fever, and anaemia were significantly concurrent in the interactions between malaria and HIV-infections.

**Table 4:** **Demographic, clinical and laboratory test characteristics by clinical phenotype of malaria and HIV infections in pregnant women**

	**Malaria alone (N = 227)**	**HIV alone (N = 6)**	**Malaria and HIV (N = 19)**	**None (N = 80)**	**P-value**
***Demographic Characteristics***					
**Maternal age (years)**					
< 20	13 (5.8%)	0 (0.0%)	1 (5.3%)	7 (8.9%)	
20–24	51 (22.6%)	0 (0.0%)	7 (36.8%)	17 (21.5%)	
> = 25	132 (71.6%)	6 (100.0%)	11 (57.9%)	55 (69.6%)	0.5
**Marital status**					
Married spouses of soldiers	194 (87.4%)	5 (50.0%)	15 (79.0%)	63 (80.8%)	
Single women as parents of soldiers	28 (12.6%)	5 (50.0%)	4 (21.0%)	15 (19.2%)	0.04
**Occupation, (%)**					
Others with income	15 (6.6%)	0 (0.0%)	4 (21.1%)	18 (22.5%)	
Housewives without income	211 (93.4%)	6 (100.0%)	15 (79.0%)	62 (77.5%)	0.001
**Number of previous pregnancies, (%)**					
Primigravidae	50 (22.1%)	0 (0.0%)	6 (31.6%)	21 (26.6%)	
Multigravidae	176 (77.9%)	6 (100.0%)	13 (68.4%)	58 (73.4%)	0.4
**Number of children, (%)**					
<3	114 (51.8%)	4 (66.7%)	12 (63.2%)	52 (65.8%)	
> = 3	106 (48.2%)	2 (33.3%)	7 (36.8%)	27 (34.2%)	0.1
**Number of abortion, (%)**					
Abortion	166 (73.1%)	5 (83.3%)	10 (52.6%)	54 (67.5%)	0.2
**Trimester of visit, (%)**					
First-trimester	112 (49.3%)	2 (33.3%)	10 (52.6%)	39 (48.7%)	
Second-trimester	60 (26.4%)	3 (50.0%)	5 (26.3%)	20 (25.0%)	
Third-trimester	55 (24.3%)	1 (16.7%)	4 (21.1%)	21 (26.3%)	0.9
**District of Kinshasa, (%)**					
Semi-urban for Amba	11 (4.8%)	2 (33.3%)	2 (10.5%)	6 (7.5%)	
Urban for Funa	160 (70.5%)	4 (66.7%)	13 (68.4%)	42 (52.5%)	0.005
Rural for Lukunga	56 (24.7%)	0 (0.0%)	4 (21.1%)	32 (40.0%)	
LBW, (%)	101 (44.7%)	2 (33.3%)	10 (52.6%)	5 (6.3%)	<0.001
***Examination findings, (%)***					
Fever	74 (32.6%)	2 (33.3%)	19 (100.0%)	32 (40.0%)	<0.001
Body aches	24 (10.7%)	0 (0.0%)	1 (5.3%)	13 (16.3%)	0.3
Headaches	33 (14.5%)	1 (16.7%)	4 (21.1%)	11 (13.8%)	0.8
Digestive disorders	24 (10.6%)	0 (0.0%)	2 (10.5%)	9 (11.3%)	0.8
Nausea and vomiting	17 (7.5%)	1 (16.7%)	1 (5.3%)	6 (7.5%)	0.8
Respiratory infection	89 (39.2%)	5 (83.3%)	7 (36.8%)	31 (38.7%)	0.1
Bleeding	18 (7.9%)	0 (0.0%)	0 (0.0%)	5 (6.3%)	0.5
Placenta previa	20 (8.8%)	0 (0.0%)	3 (15.8%)	7 (8.7%)	0.6
Urinary tract infection	147 (64.8%)	6 (100.0%)	8 (42.1%)	61 (76.2%)	0.008
***Laboratory Tests, (%)***					
Anemia (haemoglobin < 11 g/dL)^4^	74 (32.6%)	3 (50.0%)	13(68.4%)	22 (27.5%)	0.004

## Discussion

Pregnancy is associated with increased susceptibility to malaria in areas where malaria is endemic [14,15]. The control of malaria and HIV in pregnant sub-Saharan African women has, however, been particularly challenging [16]. However, rare epidemiological data have been recorded in the DRC, where *P. falciparum* malaria is highly endemic [11]. This paper describess the prevalence of malaria and HIV infections of pregnant women whose peripheral blood smears were tested.

### Malaria infection

The proportion of malaria infection in pregnancy in this study was 74.1% in Kinshasa where malaria transmission is perennial and represents a substantial burden for the local population [11]. This prevalence value is relatively consistent with those found in previous studies. However, there is a large range of previously reported prevalence values. For example, estimates of malaria in pregnancy range from 6.8% in Calabar (Nigeria) to 57.5% in Libreville (Gabon) [17,18]. The applicability of these prevalence rates is limited and muss be interpreted with caution since several articles investigating the prevalence of infection disease have cited the lack of national representation as a weakness. The results of this study revealed consistently higher association between malaria and occupation. Further evidence of the relationships between occupation and malaria infection were found in a study with a significantly higher prevalence of malaria parasites in a low socio-economic status occupational category [19-21].

By contrast with several study, fever was not significantly associated with malaria infection in the following study during pregnancy despite the fact that in endemic areas asymptomatic *Falciparum* infections are frequent in adults [21-23]. Fever alone remains a poor discriminator of malaria infection, suggesting that all fevers should be tested to confirm or refute the role of malaria in the febrile presentation [22]. This result may be due to some comorbidity.

### HIV-infection

The prevalence of HIV infection was 7.5% in the present study. This result is higher than the previous 4.2% reported in DRC by UNAIDS in 2003 [12]. A number of factors that facilitate further the spread of HIV in DRC included ignorance which refers to the lack of information, fear and stigma, the flux of refugees and soldiers, scarcity and high cost of safe blood transfusions in rural areas, few HIV testing sites, and low availability of condoms outside Kinshasa [12].

### The impact of HIV/AIDS and Malaria in pregnant women

The prevalence of HIV infection was associated to malaria in 5.7% (Figure 2). Malaria prevalence increased considerably in HIV-infected women to reach 76.0%. In areas with stable malaria transmission like DRC, HIV increases the risk of malaria infection and clinical malaria in adults, especially in those with advanced immunosuppression. In addition, HIV-infected adults are at increased risk of complicated and severe malaria and death [3-9,17,23].

Every year, approximately 25 million women become pregnant in sub-Saharan Africa, where malaria is endemic, and are at increased risk of infection with *P. falciparum*, particularly in their first two pregnancies [23]. Interactions between the two infections can have serious consequences, particularly for pregnant women. HIV-infected pregnant women who become infected with malaria are at increased risk of all the adverse outcomes of malaria in pregnancy. Co-infected pregnant women are more likely to have symptomatic malaria infections, anaemia, placental malaria infection, and low birth weight. Epidemiological studies assessing the impact of placental malaria on mother-to-child transmission of HIV have thus far been inconsistent [3-9,17,23].

### Risk factors of HIV infection

On multivariate analysis, HIV-infection was significantly associated with fever, anaemia, placenta previa, marital status, and district of residence. This finding characterized the impact of anaemia and fever related to *P. falciparum* malaria and HIV infection in pregnant women. It has been shown that many studies in Africa shows that co-infection with HIV influences the burden of malaria in pregnancy. HIV increases the degree to which malaria is associated with severe anemia and low birth weight beyond the effect of HIV itself on these outcomes. HIV also puts women of all gravidities at risk for placental infection with malaria, not only women in their first or second pregnancy [3-5,7-9,15,23]. In addition, higher HIV prevalence rates exceeding sometime 40% have been reported among pregnant women in malaria endemic region of Africa [24]. Unfortunately, the analysis of this study did not show a significant association between malaria and HIV infections (p = 0.8). This can be explained by the low number of HIV-infected patients.

### Low birth weight

LBW was highly associated to malaria infection in pregnant women. It is estimated that in areas where malaria is endemic, around 19% of infant LBWs are due to malaria and 6% of infant deaths are due to LBW caused by malaria [25,26].

### Limitations

Although the present study has yielded some important preliminary findings in regard of malaria and HIV infections in pregnant women in DRC, its design is not without flaws. A number of caveats need to be noted regarding the present study. First, as a cross-sectional design, it does not allow us to make any causal inferences from these findings. Second limitation concerns the restriction of the inclusion criteria and the absence of real follow-up due to the difficulty to enroll patients. Third, there were a relatively small number of HIV-infected pregnant women. While 332 patients were initially enrolled, only 5.7% were infected. In that case, it was relevant to examine the association between HIV-infected women and uninfected women in depth. Furthermore, the lack of unmeasured factors may bias these results. Another limitation was the challenge to collected data. Most women in this study were uneducated and were embarrassed to answer questions. In other cases, data in registers were absent or poorly reported.

### Clinical implications and perspectives of Public Health for Malaria in pregnancy

The present findings will impact on clinical practice and public health related to malaria in pregnant women. In obtaining as much relevant socioeconomic information (income, poor nutrition, residence, indoor pollution, HIV-infection, tuberculosis) as possible, present results will help to understand the pathophysiology and to apply the epidemiology evidence in clinical practice and public health. The consequences of socioeconomic deprivation on pregnancy outcomes are anaemia and low birth weight [27]. Policy makers are invited to improve social status and income levels of pregnant women married to soldiers at curbing the high burden of malaria itself and malaria adverse (anaemia and low birth weight). As recommended by the WHO, [28] the United Nations Joint Program programme on HIV/AIDS and different authors, future Congolese Studies will assess effectiveness of co-trimoxazole to prevent malaria in HIV-positive pregnant women [29,30].

Prevention of malaria in Congolese pregnant women might rely on intermittent preventive treatment with sulphadoxine-pyrimethamine, which reduces higher probability of placental parasitaemia, anaemia, and low birth weight [31,32]. Thus, good clinical practice will avoid IPT-SP-related resistance and initiation of IPT-SP before 16 weeks of pregnancy [33]. Furthermore, co-trimoxazole provides coverage for opportunistic HIV-associated infections and anti-malarial activities [34].

## Conclusions

There is evidence of high prevalence of malaria and HIV infections in Congolese pregnant women. Prevention and surveillance of infectious diseases progression are needed to reduce the incidence of malaria and HIV infections in pregnant women in DRC by improving better management and public health panel control. Qualitative research should be encouraged to obtain more in depth, locally-relevant, and descriptive data examining the impact of infectious diseases in DRC.

## Abbreviations

UPC: Protestant University of Congo
HIV: Human Immunodeficiency Virus
DRC: Democratic Republic of Congo
UNAIDS: Joint United Nations Programme on HIV/AIDS
HMRK: Camp Kokolo Military Hospital
PMTCT: Prevention of mother-to-child transmission of HIV
SD: Standard deviation
LBW: Low birth weight
